# TRIM8 Blunts the Pro-proliferative Action of ΔNp63α in a p53 Wild-Type Background

**DOI:** 10.3389/fonc.2019.01154

**Published:** 2019-11-05

**Authors:** Mariano Francesco Caratozzolo, Flaviana Marzano, Daniela Isabel Abbrescia, Francesca Mastropasqua, Vittoria Petruzzella, Viola Calabrò, Graziano Pesole, Elisabetta Sbisà, Luisa Guerrini, Apollonia Tullo

**Affiliations:** ^1^Institute of Biomembranes, Bioenergetics and Molecular Biotechnologies, National Research Council (CNR), Bari, Italy; ^2^Institute for Biomedical Technologies, National Research Council (CNR), Bari, Italy; ^3^Dipartimento di Scienze Mediche di Base, Neuroscienze e Organi di Senso, Scuola di Medicina e Chirurgia, Università degli Studi di Bari “Aldo Moro”, Bari, Italy; ^4^Department of Biology, University of Naples Federico II, Naples, Italy; ^5^Department of Biosciences, Biotechnology and Biofarmaceutics, University of Bari “Aldo Moro”, Bari, Italy; ^6^Department of Biosciences, Università degli Studi di Milano, Milan, Italy

**Keywords:** TRIM8, p53, ΔNp63α, chemoresistant tumors, caspase-1, proteasome

## Abstract

The p53 gene family network plays a pivotal role in the control of many biological processes and therefore the right balance between the pro-apoptotic and pro-survival isoforms is key to maintain cellular homeostasis. The stability of the p53 tumor suppressor protein and that of oncogenic ΔNp63α, is crucial to control cell proliferation. The aberrant expression of p53 tumor suppressor protein and oncogenic ΔNp63α contributes to tumorigenesis and significantly affects anticancer drug response. Recently, we demonstrated that TRIM8 increases p53 stability, potentiating its tumor suppressor activity. In this paper, we show that TRIM8 simultaneously reduces the level of the pro-proliferative ΔNp63α protein, in both a proteasomal and caspase-1 dependent way, thereby playing a critical role in the cellular response to DNA damaging agents. Moreover, we provided evidence that ΔNp63α in turn, suppresses TRIM8 gene expression by preventing p53-mediated transactivation of TRIM8, therefore suggesting the existence of a negative feedback loop. These findings indicate that TRIM8 exerts its anticancer power through a joint action that provides on one hand, the activation of the p53 tumor suppressor role, and on the other the quenching of the oncogenic ΔNp63α protein activity. The enhancement of TRIM8 activity may offer therapeutic benefits and improve the management of chemoresistant tumors.

## Introduction

p63 proteins are key transcription factors belonging to the *TP53* gene family. They are involved in cell growth, proliferation, apoptosis, and differentiation, playing an essential role in epithelial stem cell biology and development (1). Alternative promoter usage of *TP63* gene results in two main groups of proteins: the TA isoforms, which contain an N-terminal Trans Activation domain (TA) and the ΔN isoforms, which lack it (2, 3). For this reason, the ΔN isoforms were initially thought to act as dominant-negative isoforms until a second TA domain (TA2) was identified that accounts for their transactivation potential (4). Both TA and ΔN isoforms can be alternatively spliced to generate different carboxy-terminal proteins, including α, β, γ, δ, and ε isoforms (3). The TAp63 isoforms possess a p53-like anti-oncogenic activity and due to their potent pro-apoptotic activity are expressed at very low levels and have a relatively short half-life, whereas the pro-proliferative ΔNp63 proteins are much more stable than TAp63. TA and ΔNp63 proteins degradation is mostly proteasome-dependent and regulated by several distinct post-translational modifications, namely phosphorylation, ubiquitylation, ISGylation, and SUMOylation (5–9).

*In vitro* and *in vivo* evidences support the oncogenic role of the N-terminally truncated ΔNp63α isoform (10–12). Indeed, unlike *p53*, the *p63* gene is rarely mutated in human cancers and ΔNp63α is overexpressed in different types of tumors indicating that it provides a selective growth advantage to cancer cells. Enhanced expression of ΔNp63α is now considered a diagnostic marker and an indicator of poor prognosis (13–16). Moreover, in Squamous Cell Carcinoma (SCC), where ΔNp63α drives proliferation and blocks apoptosis, it has recently been shown that the Fibroblast Growth Factor Receptor 2 (FGFR2) is the crucial mediator of ΔNp63 oncogenic functions (17).

ΔNp63α can function both as a transcriptional activator and as a transcriptional repressor of several genes within the p53 network by simply preventing p53 occupancy at the shared p53-Responsive Elements (p53-RE) (18, 19). Accordingly, overexpression of ΔNp63α in SCCs shuts down the p53-driven transcriptional program. Therefore, a balance between p53 and ΔNp63α protein levels is pivotal to control cell proliferation, death, and differentiation.

The functional cross-talk between p53 and Tripartite motif (TRIM) E3 ubiquitin ligases that can lead to an increased or decreased degradation of p53, is known to play a key role in tumor progression and chemoresistance (20).

In particular, we have recently demonstrated that the human TRIM8 protein, is able to potentiate p53 tumor suppressor activity (21). TRIM8 protein, as all the members of this family, is characterized by the presence of a RING domain, one or two B-box motifs and a coiled-coil region. TRIM8 contains a Nuclear Localization Signal (NLS) and localizes in nuclear structures not yet characterized, whose formation is dependent on coiled-coil and C-terminus domains, suggesting that these domains provide the protein-protein interface for the recruitment of other proteins to the subcellular compartments. TRIM8 was reported to be a tumor suppressor in glioma and renal cell carcinoma (22–24). In stress condition, TRIM8 interacts with p53 tumor suppressor protein displacing MDM2 binding to p53, thus resulting in p53 stabilization and G1 cell cycle arrest (21).

Consistently with our previous findings and given the structural and functional relationship between p53 and p63, we hypothesized that TRIM8 could also be involved in the control of ΔNp63α protein stability.

Here we show evidence that TRIM8 promotes ΔNp63α destabilization both in a Caspase 1-dependent and proteasomal ways, but only in a functional p53 background. Reduction of TRIM8 cellular levels results in an increase of ΔNp63α stability, promoting a strong boost in cell proliferation and increased chemoresistance. Taken together, our data indicate that TRIM8 simultaneously increases p53 stability and reduce the level of the pro-proliferative ΔNp63α protein, thereby playing a critical role in the cellular response to DNA damaging agents.

## Materials and Methods

### Cells, Drugs and Treatments

The human embryonic kidney 293-T-Rex CAT and 293-T-Rex ΔNp63α cells were generated and cultured as reported in Sbisà et al. (25). Briefly, the human embryonic kidney Flp-In T-Rex-293 cell line from Invitrogen™ was used to generate stable ΔNp63α and CAT (Chloramphenicol acetyltransferase) as control expression cell lines. The Flp-In T-Rex-293 cell line contains two stably, independently integrated plasmids, which allowed us to integrate the cDNAs corresponding to ΔNp63α and CAT into a transcriptionally active region of the genome. The expression of ΔNp63α and CAT was induced by the addition of tetracycline to the culture medium.

The human colon cancer cell lines HCT116p53wt and HCT116p53^−/−^, the human lung carcinoma H1299 cell line, the human breast cancer MCF7 cell line, the human osteosarcoma U2OS and human keratinocyte HaCaT cell lines were all cultured in Dulbecco's modified Eagle's medium (D-MEM) plus 10% fetal bovine serum (FBS), L-Glutamine (2 mM), penicillin (100 U/ml), and streptomycin (100 μg/ml) at 37°C, 5% CO_2_.

Nutlin-3 10 μM (Cayman), Cisplatin 7.5 μM (Sigma), and UV rays (20 J/m^2^) were used for 24 h, in order to induce cell cycle arrest. Cicloheximide (CHX) (Sigma) was used at the final concentration of 100 μg/ml. MG132 (Sigma) was used for 4 h at the final concentration of 10 μM. Z-VAD-FMK (Sigma) was used for 24 h at the final concentration of 20 μM.

**TRIM8-shRNAs** (TR300821-Origene™):

TI303277) TGATAAGACGGAGGATGTCAGCTTCATGA
TI303278) AACCTGAAGCTCACCAACATCGTGGAGAA
TI303279) TAAGATCGGCCACCTGAACTCCAAGCTCT
TI303280) CGCAAGATTCTCGTCTGTTCTGTGGACAA;

**p53 shRNAs** (TR320558-Origene™):

TI379448) CAGCCAAGTCTGTGACTTGCACGTACTCC
TI379449) CCGGACGATATTGAACAATGGTTCACTGA
TI379450) CTCCTCAGCATCTTATCCGAGTGGAAGGA
TI379451) CTCAGACTGACATTCTCCACTTCTTGTTC

**Human p63 siRNA** (Dharmacon): CGACAGTCTTGTACAATTT.

### Transfections

Cells were plated 24 h before transfections. At the time of transfections (70–80% cell confluence), 200 μl of DMEM medium without serum were incubated with Trans-LT1 Mirus transfection reagent (Tema Ricerca) for 5 min at room temperature. Then, the recombinant vectors or their control empty version were added to the medium containing the transfection reagent and incubated at room temperature for 20 min and subsequently added to the cell cultures for 48 h.

### Cell Proliferation Assays by MTT Reduction

2 × 10^5^ cells were plated in six-well plates. After transfections and/or treatments, 250 μl of MTT solution (final concentration 0.5 μg/ml) was added to the cells for 4 h at 37°C. The medium was then removed and the reduced blue formazan crystals were suspended in isopropanol prior to reading the absorbance at 580 nm.

### Protein Extraction and Western Blot Analysis

Cells were plated in 100 mm culture dishes at a density of 1 × 10^6^ cells/ml. After treatments, cells were lysed, and proteins were extracted as previously described (25). Briefly, cells were lysed with RIPA buffer [50 mM Tris-HCl pH 7.5, 150 mM NaCl, 1% Nonidet P40, 0.5% sodium deoxycholate, 0.1% SDS, protease inhibitors cocktail tablets (Roche)] for 1 h on ice. Then the lysates were clarified by centrifugation at 13,200 × g at 4°C, aliquoted and stored at −20°C. Twenty micrograms of the total proteins in 2X SDS-PAGE sample buffer were heated at 95°C for 2 min and submitted to 12% SDS-PAGE. Separated proteins were electroblotted onto Nitrocellulose filter.

For immunoblotting, the following primary antibodies were used: p53 specific DO-1 (Santa Cruz, California, USA 1:300), p63 specific H-137 (Santa Cruz, California, USA 1:500), TRIM8 specific C-20 (Santa Cruz, California, USA 1:200), Anti-FLAG (Sigma, 1:2000), Anti-HA (Bethyl Laboratories, 1:1000), Anti-MDM2 (Calbiochem, Ab-2 2A10 1:200) Anti-Actin Ab-1 antibodies kit (Calbiochem, 1:2000). Bound primary antibodies were visualized using LumiLight Western Blotting Substrate (Roche™) on a UVITEC Cambridge Camera. Each experiment was repeated three times, one representative immunoblot of which is presented in the figures. In Supplementary figures is reported the densitometry analyses representing the average of three independent biological replicates with the standard deviations.

### Co-immunoprecipitation and Western Blot Analysis

Co-Immunoprecipitation experiments were performed by lysing cells in RIPA buffer with 10% glycerol in order to stabilize protein-protein interactions. Protein complexes were then immunoprecipitated using appropriate antibodies. Complexes were analyzed by western blotting using appropriate antibodies, anti-p53 DO-1 (Santa Cruz Biotechnology), anti-p63 H-137 (Santa Cruz Biotechnology), and anti-FLAG (Sigma). Bound primary antibodies were visualized using Lumi-Light Western Blotting Substrate (Roche™) on a UVITEC Cambridge Camera.

### RNA Extraction, Reverse Transcription and qRT-PCR Analysis

Total RNA was extracted from cell lines using the RNeasy Plus Mini kit (Qiagen®) and according to the manufacturer's instructions. Purified RNA was then quantified using the NanoDrop™ 1000 Spectrophotometer (Thermo Scientific) and RNA quality was determined by running aliquots on the 2100 Bioanalyzer (Agilent Technologies).

Reverse transcription of 500 ng of total RNA was performed using QuantiTect® Reverse Transcription kit (Qiagen®), according to the manufacturer's instruction. The real-time PCR reactions were performed on Applied Biosystems™ 7900HT, as described by the manufacturer. The reaction mixtures contain 2x TM Master Mix Buffer, PDAR System Target 20x FAM (TaqMan® Gene Expression Assays), 1 μl cDNA template and water. Cycle threshold (Ct) values were obtained graphically, automatically by the instrument for TRIM8, p21 (also known as CDKN1A), ADA, CCND3, and FbW7. The Normfinder applet for Microsoft Excel was used to determine the most stable housekeeping gene for MCF-7 and HCT116 cells used in this study from a panel of three reference genes (ACTB, GAPDH, and HPRT1). Normfinder analyses indicated that the glyceraldeyde 3P-dehydrogenase (GAPDH) was the most stable housekeeping gene in samples used in qRT-PCR experiments and therefore it was used as our internal standard in qPCR experiments. Reactions without cDNA were included as negative control. The relative gene expression for each experiment was calculated using the non-treated sample as a calibrator.

The data reported represent the average of at least three independent experiments and are shown with their standard deviations. In this study, a *p*-value of <0.05 was considered to be statistically significant.

### Chromatin Immunoprecipitation (ChIP) Assay

293 T-rex CAT and 293 T-rex ΔNp63α cells were treated as previously described in Sbisà et al. (25).

Twenty-four hours after induction, proteins were crosslinked to DNA in living nuclei and chromatin immunoprecipitation assay was performed as described in D'Erchia et al. (26).

Five micrograms of the following antibodies were used: p63 antibody H-137 (Santa Cruz), acetylated H4-histone antibody (Upstate™) or unrelated control anti-Flag antibody (Sigma).

DNA fragments were analyzed by PCR using specific primers for the TRIM8-p53RE1 identified in the TRIM8 intron 1.

### Luciferase Reporter Assay

2 × 10^5^ human p53-null H1299 cells were plated in 6-well plates 24 h before transfection (60–80% confluency).

pGL-3 basic recombinant vector containing the p53-RE1 of the TRIM8 gene was transfected together with the empty pcDNA_3_ expression vector or with pcDNA_3_ containing wt ΔNp63α, or its mutant version ΔNp63αR279Q, plus pRLSV40 (Promega).

Transient reporter assays were performed as previously described in D'Erchia et al. (26). Forty-eight hours after transfection, cells were lysed in Passive Lysis buffer 1X (Promega) and the luciferase assay was performed using the Dual Luciferase assay system (Promega), according to the manufacturer's instructions.

Transfection efficiency was determined by renilla activity. The data reported represent the average of at least three independent experiments and are shown with their standard deviations.

### Cell Cycle Analysis

After treatments, the total cell population, including floating and adherent cells, was harvested, washed twice with 1 × PBS, treated with 6,25 μg/ml RNase A, 50 μg/ml propidium iodide (PI), NP40 0.1% at room temperature for 1 h. The cells were analyzed in a FACScalibur; cell cycle and apoptosis analyses were performed using ModFit analysis software (Becton Dickinson).

### Statistical Analysis

Results in graphs represent the average of at least three independent experiments and are all expressed as mean ± standard deviation.

Statistical analysis was performed by using Student's *t*-test. *P* < s0.05 was considered to be statistically significant.

Imaging data were analyzed in the program Image J.

## Results

### TRIM8 Induces the Destabilization of the Oncogenic ΔNp63α Protein

**Figure 1:**
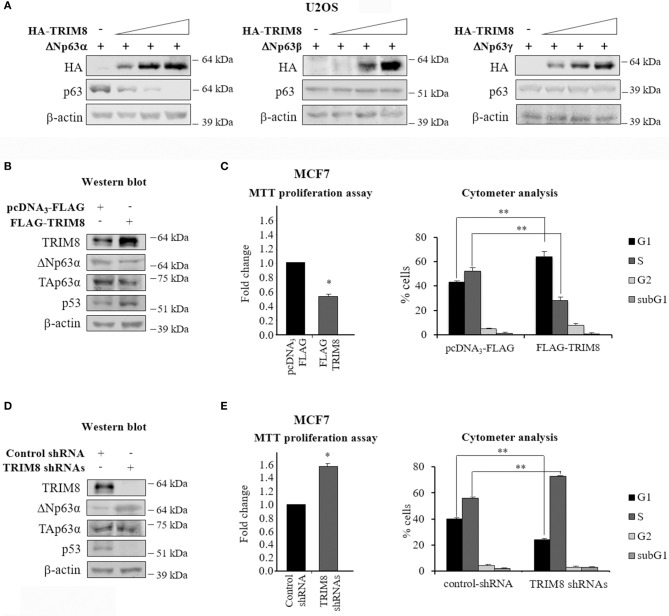
TRIM8 induces ΔNp63α destabilization. **(A)** U2OS cells were transiently co-transfected with ΔNp63α, ΔNp63β, or ΔNp63γ constructs (20 ng) and increasing amount of HA-TRIM8 expression plasmid (10, 20, and 40 ng). Western Blot (WB) analysis was performed on cells extracts with the indicated antibodies and β-actin was used as loading control. **(B,D)** WB of the indicated proteins in MCF7 cells transfected with pcDNA_3_-FLAG control vector, pcDNA_3_-FLAG-TRIM8, unspecific shRNA (control), or four specific TRIM8-shRNAs. WB of β-actin was conducted as loading control. **(C,E)** Cell proliferation was measured by MTT reduction in MCF7 cells 48 h after transfection with pcDNA_3_-FLAG control vector, pcDNA_3_-FLAG-TRIM8, unspecific shRNA (control), or four specific TRIM8-shRNAs. Data are shown as the average with a standard deviation of three independent experiments (*p < 0.05). Flow cytometric analysis of MCF7 cells transfected with pcDNA_3_-FLAG control vector, pcDNA_3_-FLAG-TRIM8, unspecific shRNA (control), or four specific TRIM8-shRNAs. Data are shown as the average with standard deviation of three independent experiments (**p < 0.01).

In this study, we sought to investigate the effects of the TRIM8 protein on ΔNp63 isoforms and in particular, on the oncogenic ΔNp63α, the main p63 isoform involved in cancer development. To this aim, TRIM8 and p63 isoforms were overexpressed in U2OS cells, bearing a low level of endogenous p63. As shown in Figure 1A and Supplementary Figure 1A, TRIM8 induced a dramatic reduction of exogenously transfected ΔNp63α but not ΔNp63β or ΔNp63γ. The same result was obtained on endogenous ΔNp63α in MCF7 cells (Figure 1B and Supplementary Figure 1B). Moreover, as expected, TRIM8 overexpression induced p53 stabilization (21), but was ineffective on TAp63α (Figure 1B and Supplementary Figure 1B). Functional assays (MTT reduction and cytofluorimetric analysis) showed that the increase of TRIM8 protein levels resulted in a decrease of cell proliferation with the arrest of the cells in G1 (Figure 1C). The percentage of apoptotic cells, as expected, was insignificant (subG1 in Figure 1C) (21). Conversely, TRIM8 depletion, by specific short hairpin RNAs (shRNAs) resulted in an increase of cell viability and entry in S-phase (Figures 1D,E). Accordingly, we found an increase of ΔNp63α and a reduction of p53 levels by immunoblot analysis upon TRIM8 depletion (Figure 1D and Supplementary Figure 1C).

**Figure 2:**
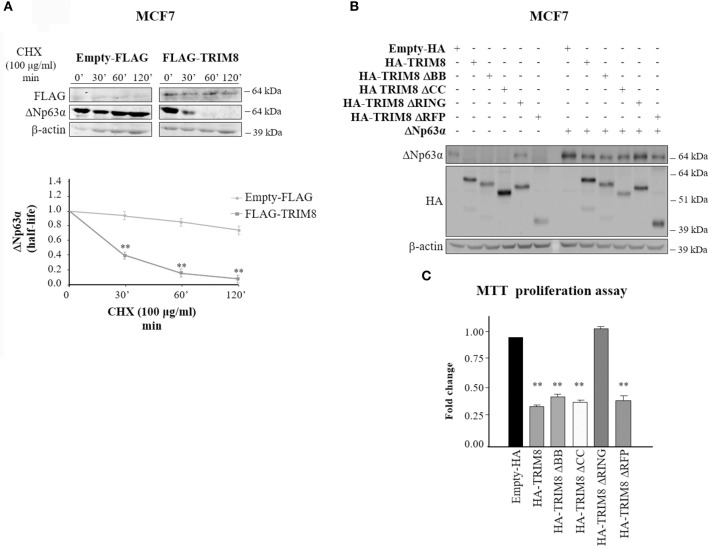
The RING domain of TRIM8 is necessary to mediate ΔNp63α destabilization. **(A)** Half-life of endogenous ΔNp63α protein in MCF7 cells transfected with empty pcDNA_3_-FLAG or pcDNA_3_-FLAG-TRIM8 expression vectors for 48 h and treated with cycloheximide (CHX) (100 μg/ml) for the indicated times (minutes). Data are shown as the average with standard deviation of three independent experiments (**p < 0.01). **(B)** Both the endogenous and ectopic levels of ΔNp63α protein were assessed by immunoblotting with the indicated antibodies in the MCF7 cell line transfected for 48 h with pcDNA_3_-HA control vector, pcDNA_3_-HA-TRIM8, or with TRIM8-deletion mutants, lacking the two B-boxes (TRIM8-ΔBB), the coiled coil domain (TRIM8-ΔCC), the RING domain (TRIM8-ΔRING), or the RFP domain (TRIM8-ΔRFP). **(C)** Cell proliferation was measured by MTT reduction in the MCF7 cell line, expressing endogenous ΔNp63α and transfected for 48 h with pcDNA_3_-HA control vector, pcDNA_3_-HA-TRIM8, or with TRIM8-deletion mutants, lacking the two B-boxes (TRIM8-ΔBB), the coiled-coil domain (TRIM8-ΔCC), the RING domain (TRIM8-ΔRING), or the RFP domain (TRIM8-ΔRFP). Data are shown as the average with standard deviation of three independent experiments (**p < 0.01).

To quantify the effect of TRIM8 on ΔNp63α stability we performed a time-course experiment using cycloheximide (CHX) in MCF7 cells transfected with pcDNA_3_ empty vector (control) or pcDNA_3_-TRIM8. As shown in Figure 2A and Supplementary Figure 2A, TRIM8 dramatically decreased ΔNp63α protein half-life. To identify which domain of TRIM8 was responsible for ΔNp63α destabilization, we transfected constructs encoding different TRIM8 deletion mutants lacking the RING domain (TRIM8-ΔRING), the B-boxes domain (TRIM8-ΔBB), the coiled-coil domain (TRIM8-ΔCC) or the RFP (RING-Finger Protein-like) domain (TRIM8-ΔRFP) in control and ΔNp63α overexpressing MCF7 cells. Immunoblot analysis revealed that only the TRIM8-ΔRING mutant was unable to reduce endogenous and overexpressed ΔNp63α protein levels as well as to promote p53 stabilization and activation and MDM2 degradation, as previously reported (21) (Figure 2B, compare lane 1 with lane 5 and lane 7 with lane 11 and Supplementary Figure 2B). Accordingly, TRIM8-ΔRING mutant did not cause the reduction of MCF7 cell proliferation thus demonstrating that the RING domain is absolutely required for TRIM8-dependent control of ΔNp63α stability and activity (Figures 2B,C). Moreover, following TRIM8 transfection, the expression of two ΔNp63α target genes associated to cell cycle progression, ADA and CCND3 (19, 27) dramatically dropped (Supplementary Figures 3A,B).

Altogether, these data strongly demonstrate that TRIM8 induces the destabilization of the oncogenic ΔNp63α protein and that the RING domain of TRIM8 is necessary to mediate this process.

### p53-Dependent Effect of TRIM8 on ΔNp63α Destabilization

**Figure 3:**
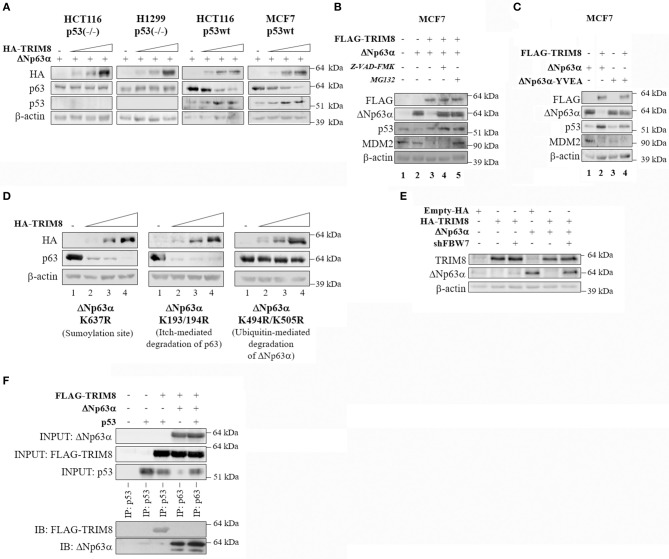
p53-dependent effect of TRIM8 on ΔNp63α destabilization. **(A)** p53 null HCT116-p53(–/–) and H1299 cells and p53wt HCT116-p53wt and MCF7(p53wt) cells were transiently co-transfected with ΔNp63α (20 ng) and an increasing amount of HA-TRIM8 expression plasmid (10, 20, and 40 ng). WB analysis was performed on cells extracts with the indicated antibodies. **(B)** MCF7 cells were transiently transfected with the indicated recombinant vectors for 48 h and treated with the caspases-inhibitor Z-VAD-FMK (20 μM for 24 h) or with the proteasome inhibitor MG132 (10 μM for 4 h). WB analysis was performed on cells extracts with the indicated antibodies. The endogenous level of ΔNp63α in MCF7 cells is shown in lane 1; transfected ΔNp63α (shown in lane 2) is about 24 folds higher than endogenous level. **(C)** MCF7 cells were transiently co-transfected with pcDNA_3_-FLAG or pcDNA_3_-FLAG-TRIM8 constructs together with ΔNp63α or mutant ΔNp63α YVEA for 48 h. WB analysis was performed on cells extracts with the indicated antibodies. **(D)** U2OS cells were transiently co-transfected with mutant ΔNp63α constructs (K637R; K193/194R; K494/505R) (20 ng) and increasing amount of HA-TRIM8 expression plasmid (10, 20, and 40 ng). WB analysis was performed on cells extracts with the indicated antibodies. **(E)** U2OS cells were transiently co-transfected with the indicated recombinant vectors for 48 h. WB analysis was performed on cells extracts with the indicated antibodies. **(F)** Co-immunoprecipitation of ΔNp63α and TRIM8. HCT116-p53(–/–) were co-transfected with the indicated plasmids and treated with MG132 for 4 h at the final concentration of 10 μM. Cell lysates were immunoprecipitated with anti-p63 or anti-p53 (control). The immunoprecipitated complexes were analyzed by western blotting with the indicated antibodies.

To evaluate whether the effect of TRIM8 on ΔNp63α stability was p53 dependent, we investigated the effect of TRIM8 overexpression on ΔNp63α stability in p53-null cells (HCT116 p53^−/−^, H1299 p53^−/−^) or p53 wild type cell lines (HCT116 p53^+/+^, MCF7), expressing ΔNp63α, and TRIM8 proteins (Supplementary Figure 3C). Interestingly, TRIM8-dependent ΔNp63α destabilization was observed only in a p53 wild type cells and not in p53-null and in p53-mutated background (Figure 3A and Supplementary Figures 4A,B), indicating that ΔNp63α destabilization by TRIM8 was p53-dependent. Moreover, we observed that in MCF7 cells (expressing high ΔNp63α protein levels), TRIM8 overexpression did not destabilize ΔNp63α when p53 was silenced with specific p53-shRNA (Supplementary Figure 4C) thus definitely confirming the importance of p53 in this process.

Next, we tested whether TRIM8-mediated ΔNp63α destabilization occurred by a caspase or proteasome-dependent pathway. MCF7 cells were co-transfected with ΔNp63α and TRIM8 and treated either with the Caspase Inhibitor Z-VAD-FMK or with the proteasome inhibitor MG132. As shown in Figure 3B and Supplementary Figure 5A, both MG132 and Z-VAD-FMK treatments prevented ΔNp63α degradation upon TRIM8 overexpression (compare lane 3 with lane 4 and 5, respectively). Remarkably, TRIM8 induced proteasome-dependent MDM2 destabilization (compare lane 2 with lanes 3 and 5 in Figure 3B and Supplementary Figure 5A). Furthermore, the mutant ΔNp63α YVEA in which the Caspase-1 site is mutated (28), was resistant to TRIM8 overexpression, endorsing the hypothesis that TRIM8 induces ΔNp63α destabilization by both the proteasome and caspase1-dependent pathways (Figure 3C and Supplementary Figure 5B).

Remarkably, the mutant ΔNp63α-K637R, in which the sumoylation site is mutated (5), and the mutant ΔNp63α-K193/194R, that is resistant to Itch-mediated degradation (29) were both sensitive to TRIM8 overexpression (Figure 3D and Supplementary Figure 5C). Conversely, the mutant ΔNp63α-K494R/K505R (30) was resistant to TRIM8 overexpression. These lysines have been shown to be involved in ubiquitin-mediated degradation following nuclear-cytoplasmic shuttling of ΔNp63α upon MDM2 action, suggesting that nuclear export of ΔNp63α is required for TRIM8 mediated regulation (Figure 3D and Supplementary Figure 5C). Once exported to the cytoplasm by MDM2, p63 is targeted for degradation by the Fbw7 E3-ubiquitin ligase (30). To test if FbW7 is involved in ΔNp63α degradation upon TRIM8 overexpression, we silenced FbW7 expression in U2OS by using specific sh-RNAs (30). The silencing of FbW7 was confirmed by qPCR experiments (Supplementary Figure 5D). As shown in Figure 3E and Supplementary Figure 5E, FbW7 knockdown induced a partial recovery of ΔNp63α protein levels upon TRIM8 overexpression, suggesting that Fbw7 may be involved in TRIM8-induced ΔNp63α degradation although we could not detect a direct interaction between TRIM8 and ΔNp63α by co-immunoprecipitation (Figure 3F).

### ΔNp63α Negatively Regulates TRIM8 Expression

**Figure 4:**
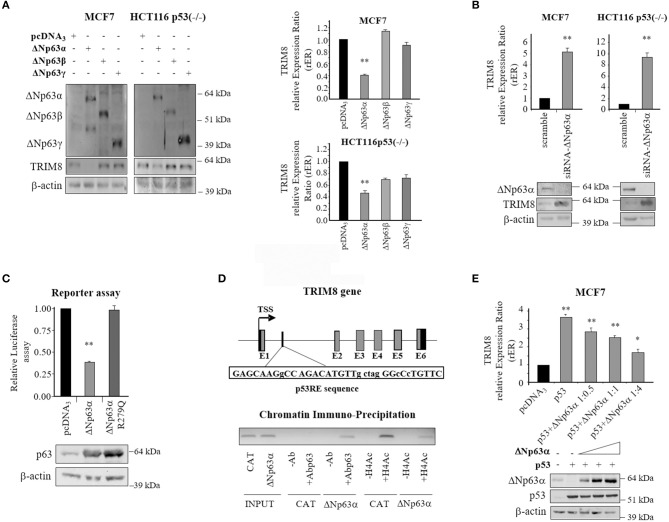
Repressive role of ΔNp63α on TRIM8 gene expression. **(A)** Western Blotting with the indicated antibodies in MCF7 and HCT116-p53(–/–) cells transfected with pcDNA_3_-control vector, pcDNA_3_-ΔNp63α, pcDNA_3_-ΔNp63β, or pcDNA_3_-ΔNp63γ. WB of β-actin was conducted as loading control. RT-qPCR of TRIM8-mRNA in MCF7 and HCT116-p53(–/–) cells transfected with pcDNA_3_-control vector, pcDNA_3_-ΔNp63α, pcDNA_3_-ΔNp63β, or pcDNA_3_-ΔNp63γ. Data are shown as the average with standard deviation of three independent experiments (**p < 0.01). **(B)** RT-qPCR of TRIM8 -mRNA in MCF7 and HCT116-p53(–/–) cells transfected with unspecific siRNA (scramble) or specific siRNA–ΔNp63α. Data are shown as the average with a standard deviation of three independent experiments (**p < 0.01). The level of endogenous ΔNp63α and TRIM8 in the same cells were measured by western blotting reported below. WB of β-actin was conducted as loading control. **(C)** Luciferase reporter assay. H1299 cells were co-transfected with plasmids expressing ΔNp63α or its mutated form (ΔNp63αR279Q) and pGL3-Basic-TRIM8-p53RE luciferase reporter. The luciferase activities were measured 48 h after transfection. Data are shown as the average with standard deviation of three independent experiments (**p < 0.01). The levels of exogenously expressed protein were controlled by western blotting. **(D)** Schematic map of the human TRIM8 genomic region containing the putative p53RE (TRIM8-p53RE) with the related sequence. Below, the *in vivo* recruitment of ΔNp63α and acetylated H4 histone to TRIM8-p53RE present in the TRIM8 gene by Chromatin-immunoprecipitation assay. DNA fragments were analyzed by PCR using specific primers. **(E)** RT-qPCR of TRIM8-mRNA in MCF7 cells transiently co-transfected with p53 and an increasing amount of ΔNp63α expression plasmid. Data are shown as the average with standard deviation of three independent experiments (*p < 0.05; **p < 0.01). WB analysis was performed on cells extracts with the indicated antibodies. WB of β-actin was conducted as loading control.

Next, we sought to determine if also the pro-oncogenic ΔNp63α isoform was able to regulate TRIM8 expression, as it has been shown for p53 (21). Thus, we transfected both MCF7 cells (p53wt) and HCT116 (p53^−/−^) cells with ΔNp63α expression vector. As shown in Figure 4A and Supplementary Figure 6A, western blot experiments and qPCR analyses demonstrated that ΔNp63α overexpression dramatically repressed TRIM8 expression in both cell lines. Conversely, ΔNp63β and γ overexpression did not influence at all TRIM8 expression in MCF7 cells, while induced only a slight decrease of TRIM8 mRNA and protein levels in p53-null HCT116. The depletion of endogenous ΔNp63α in MCF7 (p53wt) and HCT116 (p53^−/−^) cells by specific small interfering RNA (siRNA) increased TRIM8 transcript levels, indicating the possible repressive action of ΔNp63α on TRIM8 in a more physiological setting (Figure 4B and Supplementary Figure 6B).

Next we tested whether ΔNp63α was able to bind the p53-Responsive Element (RE) that we identified within intron 1 of *TRIM8* (21). Luciferase reporter assays showed that ΔNp63α overexpression repressed TRIM8 p53-RE (Figure 4C). Transcriptional repression was dependent on functional ΔNp63α since the DNA binding defective mutant ΔNp63αR279Q was unable to repress the reporter construct (31) (Figure 4C and Supplementary Figure 6C). *In vivo* binding of ΔNp63α to this p53RE was verified by Chromatin Immuno-Precipitation (ChIP) experiments in a 293T-rex stable cell line expressing ΔNp63α under the control of a tetracycline-inducible promoter. Interestingly, we found that ΔNp63α bound as well as p53 the same RE in TRIM8 gene with the opposite effect: repression for ΔNp63α and activation for p53. Indeed, the recruitment of ΔNp63α on p53RE was paralleled by a decrease in histone H4 acetylation (Figure 4D and Supplementary Figure 6D). These results further support the repressive role of ΔNp63α on TRIM8 gene expression indicating the existence of a negative auto-regulatory loop.

Based on these results we tested whether there was a competitive action between p53 and ΔNp63α on TRIM8 promoter. To this aim, p53 and ΔNp63α were transfected in MCF7 cells alone or together in different ratios. TRIM8 mRNA expression levels were evaluated by RT-qPCR. As shown in Figure 4E and Supplementary Figure 6E, when p53 was overexpressed alone, TRIM8 expression increased, but when p53 was overexpressed along with ΔNp63α, TRIM8 mRNA expression decreased along with the increase of ΔNp63α protein levels indicating that ΔNp63α may compete with p53 for the control of *TRIM8* gene expression. Similar results were obtained in HCT116 p53^−/−^ cells indicating that ΔNp63α not only competes with p53 but is able to suppress TRIM8 gene expression in a p53-independent way (Supplementary Figure 7).

### TRIM8 Deficit Impairs ΔNp63α Response to Cellular Stress

**Figure 5:**
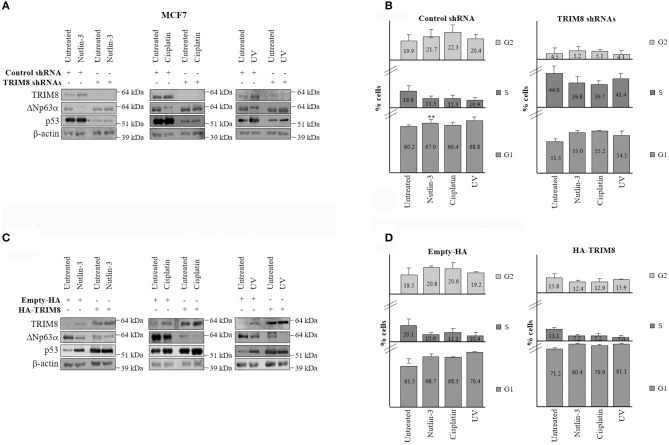
Inhibition of TRIM8 negatively affects the response to cellular stresses. **(A)** WB of the indicated proteins in MCF7 cells transfected with control unspecific shRNA or TRIM8-specific shRNAs and treated with Nutlin-3 (10 μM), Cisplatin (7.5 μM), UV rays (20 J/m^2^), or untreated cells. WB of β-actin was conducted as loading control. **(B)** Flow cytometric analysis of MCF7 cells transfected with control unspecific shRNA or TRIM8-specific shRNAs and treated with Nutlin-3 (10 μM), Cisplatin (7.5 μM), UV rays (20 J/m^2^), or untreated cells. **(C)** WB of the indicated proteins in MCF7 transfected with empty pcDNA_3_-HA or the recombinant pcDNA_3_-HA-TRIM8 vectors and treated with Nutlin-3 (10 μM), Cisplatin (7.5 μM), UV rays (20 J/m^2^), or untreated cells. WB of β-actin was conducted as control. **(D)** Flow cytometric analysis of MCF7 cells transfected with empty pcDNA_3_-HA or the recombinant pcDNA_3_-HA TRIM8 vectors, and treated with Nutlin-3 (10 μM), Cisplatin (7.5 μM), UV rays (20 J/m^2^), or untreated cells. Data are shown as the average with standard deviation of three independent experiments (**p < 0.01).

It has been widely reported that in response to UV irradiation or to chemotherapy treatments as cisplatin, which lead to p53 activation, ΔNp63α protein levels decreases (25, 32). To test the effects of TRIM8 deficit on ΔNp63α stability and activity after UV irradiation, Nutlin-3, or Cisplatin treatments, MCF7 cells were transfected with control shRNA (scramble) or specific TRIM8 shRNA and treated with UV, Nutlin-3, or Cisplatin for 24 h. Immunoblot experiments demonstrated that Nutlin-3, Cisplatin, and U.V. induced an increase in p53 and TRIM8 protein levels and a decrease of ΔNp63α protein (Figure 5A and Supplementary Figure 8A). TRIM8 was also up regulated at the transcriptional level (Supplementary Figure 8B, lanes with control shRNA). As a result, p21 mRNA levels increased (Supplementary Figure 8C) with concomitant reduction of cell proliferation (Figure 5B and Supplementary Figure 8D, lanes with control shRNA). Coherently, the expression of the ΔNp63α-target genes ADA and CCND3, decreased (Supplementary Figure 9A, lanes with control shRNA). Depletion of TRIM8 by specific shRNA caused a strong ΔNp63α stabilization (Figure 5A and Supplementary Figure 8A) followed by the reduction of p21 mRNA, the increase of ADA, CCND3 mRNA levels (Supplementary Figures 8C, 9A) with a significant increase of percentage of the cells in S-phase (16.3 vs. 48.6% in Figure 5B, untreated samples) even under Nutlin-3, Cisplatin, or U.V. treatments (Figure 5B). On the other hand, TRIM8 overexpression in cells subjected to Nutlin-3, Cisplatin, or U.V. irradiation induced ΔNp63α destabilization that was concomitant with the increase of p53 and p21 and the down-regulation of ADA, CCND3 mRNA levels (Supplementary Figure 9B) resulting in an increase of the percentage of the cells in G1-phase and the arrest of cell proliferation (Figures 5C,D and Supplementary Figures 10A,B).

Similar results were obtained also in different cell lines expressing wild type p53 as HCT116 and U2OS (Supplementary Figures 11–14).

Altogether these experiments indicate that inhibition of TRIM8 negatively affects the response of the cells to UV exposure, Nutlin-3, and Cisplatin treatments underlying the critical role played by TRIM8 in mediating the p53-dependent cell cycle arrest in response to DNA damage and that TRIM8 mediated down-regulation of ΔNp63α is essential in this context.

## Discussion

The levels of proteins such as p63 and p53 that control fundamental cellular processes, including gene expression and cell proliferation, must be critically regulated. The destabilization of p63 proteins is isoform-dependent. TAp63 isoforms are generally less stable than ΔN variants that are known to be target for regulated ubiquitination and proteolysis (33, 34). So far, several ubiquitin E3-ligases controlling p63 proteins have been identified. Nedd4 was the first E3 ligase to be identified as acting on p63 (35). Itch is a Nedd4-like ubiquitin E3 ligase that directly binds and ubiquitinates both TA- and ΔN-p63α isoforms promoting their proteasome degradation (29). WWP1 (WW domain containing E3 ubiquitin protein ligase-1), the homolog of Itch and Pirh2 (p53-induced RING-H2) can also bind both TAp63α and ΔNp63α promoting their proteasome mediated degradation (36). Following DNA damage or during keratinocyte differentiation, MDM2 binds to ΔNp63α promoting its translocation to the cytoplasm, where it is ubiquitylated by the E3 ubiquitin ligase Fbw7 and directed to proteasome degradation (30). Finally, p53 was shown to be able to associate with and target ΔNp63α into a Caspase1-dependent protein degradation pathway (28).

In our study, we demonstrate that TRIM8 overexpression induces the destabilization of ΔNp63α, but not of the β and γ isoforms, by both proteasome and caspase-dependent mechanisms. The presence of a Sterile Alpha Motif (SAM) only at the C-terminal of only the alpha isoform, suggests that this domain may be involved in the regulation of ΔNp63α stability mediated by TRIM8. Indeed the function of this domain is not limited to oligomer formation but can be variable depending on different protein interactions. On the other hand, the TAp63α isoform, despite owing the SAM domain, is resistant to TRIM8 effect, possibly due to the reduced accessibility of its three-dimensional structure (6). Further studies may help to establish if the SAM domain in combination with post-translational modifications is necessary for ΔNp63α destabilization upon TRIM8 overexpression.

**Figure 6:**
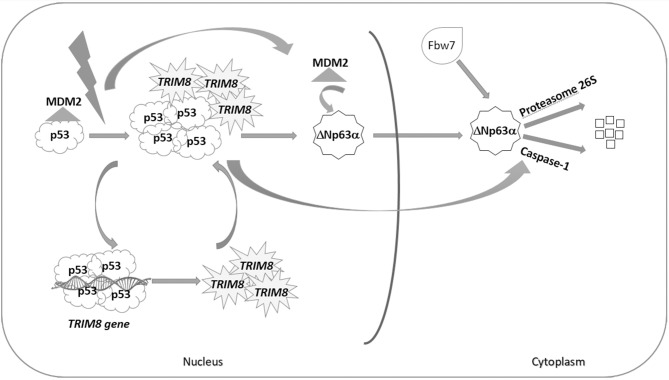
p53-dependent effect of TRIM8 on ΔNp63α destabilization. Under stress condition, p53 activates the expression of TRIM8, which in turn binds p53 and displaces MDM2 inducing p53 stabilization. TRIM8 simultaneously reduces the level of the pro-proliferative ΔNp63α protein. We could hypothesize that MDM2, before being itself degraded, could mediate ΔNp63α translocation to the cytoplasm, where proteasomal degradation mediated by the E3 ubiquitin ligase Fbw7 could occur. Moreover, TRIM8-stabilized p53 mediates ΔNp63α destabilization through the Caspase-1 pathway.

We have previously demonstrated that TRIM8 induces p53 stabilization (21) and now we show evidence that one of the roles played by TRIM8-stabilized p53 is to mediate ΔNp63α destabilization through a Caspase-1 pathway (28) (Figure 6). TRIM8 dramatically decreased the ΔNp63α half-life in a way that is similar to the effect of TRIM8 on the E3 ubiquitin ligase MDM2, the main p53 negative regulator [Figure 3B, compare lane 2 with lane 3; Caratozzolo et al. (21)]. Since TRIM8 binds p53 and displaces the binding of MDM2 to p53 (21), we could hypothesize that MDM2 itself, before being itself degraded, could mediate ΔNp63α translocation to the cytoplasm, where proteasomal degradation mediated by the E3 ubiquitin ligase Fbw7 could occur. Indeed silencing of Fbw7 induces a partial recovery of ΔNp63α protein levels upon TRIM8 overexpression, suggesting that Fbw7 is involved together with Caspase-1 in ΔNp63α destabilization (Figure 6). The hypothesis that TRIM8 may act itself as an E3 ubiquitin ligase for ΔNp63α, requires further investigation since we do not find any direct interaction between the two proteins. Anyway, we believe that these mechanisms are not redundant but rather they may ensure a quick and efficient cellular response to DNA damage stimuli that requires a rise in the p53 tumor suppressor activity and a sharp decrease of the pro-proliferative ΔNp63α protein. Indeed, interactions between p53 family members and their isoforms have a profound effect on tumorigenesis and anticancer drug response. The most intriguing are those between ΔN and TA isoforms. In cancer cells expressing high levels of ΔNp63α, the transactivation property of p53 and TAp63 isoforms, which induce cell cycle arrest and apoptosis, is suppressed by the dominant-negative action of ΔNp63α. This suppression makes cells resistant to cell cycle arrest and apoptosis, causing uncontrolled cell proliferation and tumor formation. Moreover, ΔNp63α is considered a key determinant of therapeutic response to cisplatin in tumors expressing p53, as it induces ΔNp63α degradation and stabilization of the TAp63α, proapoptotic isoform (37, 38). A consistent picture emerging from the literature asserts that certain genotoxic stress, as well as chemotherapeutic drugs, induce an apoptotic response mediated both by destabilization of the anti-apoptotic ΔN isoforms and by the stabilization of the pro-apoptotic TA isoforms. In other words, it seems that the execution of the p53-mediated cell proliferation arrest and apoptosis requires the depletion of the ΔNp63α isoform (15).

Interestingly, we provided evidence that ΔNp63α, in turn, suppresses TRIM8 gene expression by preventing p53-mediated transactivation of TRIM8. Indeed, ΔNp63α was found to function as a transcriptional repressor of several genes within the p53 network by simply preventing p53 occupancy at the shared p53-Responsive Elements (18, 19). Therefore, the aberrant expression of ΔNp63α may promote tumorigenesis by inhibiting the function of wild type p53. Cancer cells that fail to execute a p53-mediated cell cycle arrest or apoptosis develop chemoresistance, which is a major problem in cancer therapy, particularly in those tumors where p53 is present in an inactive form. Therefore, it is crucial to search key regulators that target ΔNp63α protein degradation preserving the tumor suppressor role of the TA isoforms.

In this context, the studies reported in this paper shed new light on a model mechanism by which TRIM8 protein exerts its anticancer activity through a joint action that provides on one hand the activation of the p53 tumor suppressor role and, on the other, the quenching of the oncogenic activity of the ΔNp63α protein.

In conclusion, we found that TRIM8 promotes ΔNp63α destabilization, although not by direct interaction, and only in a p53 wild type cellular background. Remarkably, we have shown that TRIM8 is able to increase the cellular responses to UV, Nutlin-3, and cisplatin in different tumor cells, thereby playing a critical role in the outcome of DNA damaging agents treatment.

Mediated strategies to enhance TRIM8 that in turn activates the p53 tumor suppressor function and blunt the oncogenic activity of ΔNp63α may offer therapeutic benefits and are likely to improve the management of chemoresistant tumors.

## Data Availability Statement

The raw data supporting the conclusions of this manuscript will be made available by the authors, without undue reservations, to any qualified researcher.

## Author Contributions

MC and AT conceived and designed the study. MC, FMar, FMas, and DA performed the experiments. MC, FMar, FMas, VP, GP, ES, LG, and AT acquired, analyzed, and interpreted the data. AT, VC, VP, GP, ES, and LG drafted and edited the manuscript. All the authors critically revised the manuscript for intellectual content. All authors approved the final version of the manuscript.

### Conflict of Interest

The authors declare that the research was conducted in the absence of any commercial or financial relationships that could be construed as a potential conflict of interest.
