# Supplementary material for: TRIM8 Blunts the Pro-proliferative Action of ΔNp63α in a p53 Wild-Type Background
Source: Front Oncol. 2019 Nov 5;9:1154. doi: 10.3389/fonc.2019.01154 (PMC6856647; doi:10.3389/fonc.2019.01154)
Supplement: Supplementary file 1 [file Data_Sheet_1.docx]

**Supplementary Figure Legends**

**Supplementary Fig. 1. (A)** Densitometry analysis of the average of 3 immunoblot experiments, one of which is presented in Figure 1A. The error bars represent the standard deviation. **(B)** Densitometry analysis of the average of 3 immunoblot experiments, one of which is presented in Figure 1B. The error bars represent the standard deviation. **(C)** Densitometry analysis of the average of 3 immunoblot experiments, one of which is presented in Figure 1D. The error bars represent the standard deviation. The relative amounts of proteins, reported in the tables and the graphics, have been calculated normalizing to β-actin and calibrating to the respective control.

**Supplementary Fig. 2. (A)** Densitometry analysis of the average of 3 immunoblot experiments, one of which is presented in Figure 2A. The error bars represent the standard deviation. **(B)** Densitometry analysis of the average of 3 immunoblot experiments, one of which is presented in Figure 2B. The error bars represent the standard deviation. The relative amounts of proteins, reported in the table, have been calculated normalizing to β-actin and calibrating to the respective control.

**Supplementary Fig. 3. (A, B)** RT-qPCR of ADA and CCND3 mRNAs in MCF7 cells transfected with empty-pcDNA3-HA, pcDNA3-ΔNp63α and pcDNA3-HA-TRIM8 plus pcDNA3-ΔNp63α expression plasmids. Data are shown as the average with standard deviation of 3 independent experiments (* p-value < 0.05; ** p-value < 0.01). WB of the indicated proteins in the same cell extracts. WB of β-actin was conducted as control. Densitometry analysis of the average of 3 immunoblot experiments, one of which is presented in the figure. The relative amounts of proteins, reported in the table, have been calculated normalizing to β-actin and calibrating to the respective control. The error bars represent the standard deviation. **(C)** The basal level of the indicated proteins was analysed by WB in HCT116(p53-/-), in H1299(p53-/-), in HCT116(p53wt) and in MCF7(p53wt). Below, it is reported the densitometry analysis of the average of 3 immunoblot experiments, one of which is presented in the figure. The relative amounts of proteins, reported in the table, have been calculated normalizing to β-actin and calibrating to the respective control. The error bars represent the standard deviation.

**Supplementary Fig. 4. (A)** Densitometry analysis of the average of 3 immunoblot experiments, one of which for cell line is presented in Figure 3A. The relative amounts of proteins, reported in the table, have been calculated normalizing to β-actin and calibrating to the respective control. The error bars represent the standard deviation. **(B)** WB of the indicated proteins in HaCat cells with both p53 alleles mutated, transfected with pcDNA3-HA control vector, pcDNA3-HA-TRIM8. WB of β-actin was conducted as control. Below, it is reported the densitometry analysis of the average of 3 immunoblot experiments, one of which is presented. The relative amounts of proteins, reported in the table, have been calculated normalizing to β-actin and calibrating to the respective control. The error bars represent the standard deviation. **(C)** WB of the indicated proteins in MCF7 cells transfected with pcDNA3-HA control vector or pcDNA3-HA-TRIM8, plus pcDNA3-ΔNp63α and specific p53-shRNAs. WB of β-actin was conducted as control. On the right it is reported the densitometry analysis of the average of 3 immunoblot experiments, one of which is presented in the figure. The relative amounts of proteins, reported in the table, have been calculated normalizing to β-actin and calibrating to the respective control. The error bars represent the standard deviation.

**Supplementary Fig. 5. (A)** Densitometry analysis of the average of 3 immunoblot experiments, one of which for cell line is presented in Figure 3B. The relative amounts of proteins, reported in the table, have been calculated normalizing to β-actin and calibrating to the respective control. The error bars represent the standard deviation. **(B)** Densitometry analysis of the average of 3 immunoblot experiments, one of which for cell line is presented in Figure 3C. The relative amounts of proteins, reported in the table, have been calculated normalizing to β-actin and calibrating to the respective control. The error bars represent the standard deviation. **(C)** Densitometry analysis of the average of 3 immunoblot experiments, one of which for cell line is presented in Figure 3D. The relative amounts of proteins, reported in the table, have been calculated normalizing to β-actin and calibrating to the respective control. The error bars represent the standard deviation. **(D)** Expression levels of Fbw7 were measured by RT-qPCR in the U2OS cells transfected with the indicated constructs. Relative expression ratios were measured respect to the sample transfected with the pcDNA_3_-empty-HA vector and normalized by the expression levels of GAPDH. Data are shown as the average with standard deviation of 3 independent experiments (** pvalue < 0.01). **(E)** Densitometry analysis of the average of 3 immunoblot experiments, one of which for cell line is presented in Figure 3E. The relative amounts of proteins, reported in the table, have been calculated normalizing to β-actin and calibrating to the respective control. The error bars represent the standard deviation.

**Supplementary Fig. 6. (A)** Densitometry analysis of the average of 3 immunoblot experiments, one of which for cell line is presented in Figure 4A. The relative amounts of proteins, reported in the table, have been calculated normalizing to β-actin and calibrating to the respective control. The error bars represent the standard deviation. **(B)** Densitometry analysis of the average of 3 immunoblot experiments, one of which for cell line is presented in Figure 4B. The relative amounts of proteins, reported in the table, have been calculated normalizing to β-actin and calibrating to the respective control. The error bars represent the standard deviation. **(C)** Densitometry analysis of the average of 3 immunoblot experiments, one of which for cell line is presented in Figure 4C. The relative amounts of proteins, reported in the table, have been calculated normalizing to β-actin and calibrating to the respective control. The error bars represent the standard deviation. **(D)** Densitometry analysis of the average of 3 ChIP experiments, one of which for cell line is presented in Figure 4D. The relative intensity of the bands, reported in the table, have been calculated normalizing to CAT and ΔNp63α INPUT controls. The error bars represent the standard deviation. **(E)** Densitometry analysis of the average of 3 immunoblot experiments, one of which for cell line is presented in Figure 4E. The relative amounts of proteins, reported in the table, have been calculated normalizing to β-actin and calibrating to the respective control. The error bars represent the standard deviation.

**Supplementary Fig. 7.** RT-qPCR of TRIM8-mRNA in HCT116(p53-/-) cells transiently cotransfected with p53 and increasing amount of _Np63α expression plasmid. Data are shown as the average with standard deviation of 3 independent experiments (** pvalue < 0.01). Densitometry analysis of the average of 3 immunoblot experiments, one of which for cell line is presented in the figure. The relative amounts of proteins, reported in the table, have been calculated normalizing to β-actin and calibrating to the respective control. The error bars represent the standard deviation.

**Supplementary Fig. 8. (A)** Densitometry analysis of the average of 3 immunoblot experiments, one of which for cell line is presented in Figure 5A. The relative amounts of proteins, reported in the table, have been calculated normalizing to β-actin and calibrating to the respective control. The error bars represent the standard deviation. **(B, C)** Expression levels of TRIM8 and p21 were measured by RT-qPCR in the MCF7 cells transfected with control unspecific shRNA or TRIM8-specific shRNAs and treated with Nutlin-3 (10 μM), Cisplatin (7.5 μM), UV rays (20 J/m^2^), or untreated cells. Relative expression ratios were measured respect to the sample transfected with the control unspecific shRNAs and normalized by the expression levels of GAPDH. Data are shown as the average with standard deviation of 3 independent experiments (* p-value < 0.05; ** p-value < 0.01). **(D)** Cell proliferation by MTT reduction assay in MCF7 transfected with control unspecific shRNA or TRIM8-specific shRNAs and treated with Nutlin-3 (10 μM), Cisplatin (7.5 μM), UV rays (20 J/m^2^), or untreated cells.

Data are shown as the average with standard deviation of 3 independent experiments (* p-value < 0.05; ** p-value < 0.01).

**Supplementary Fig. 9. (A)** Expression levels of ADA and CCND3 were measured by RT-qPCR in the MCF7 cells transfected with control unspecific shRNA or TRIM8-specific shRNAs and treated with Nutlin-3 (10 μM), Cisplatin (7.5 μM), UV rays (20 J/m^2^), or untreated cells. Relative expression ratios were measured respect to the sample transfected with the control unspecific shRNA and normalized by the expression levels of GAPDH. Data are shown as the average with standard deviation of 3 independent experiments (* p-value < 0.05; ** p-value < 0.01). **(B)** Expression levels of p21, ADA and CCND3 were measured by RT-qPCR in the MCF7 cells transfected with empty pcDNA3-HA or the recombinant pcDNA3-HA-TRIM8 vectors and treated with Nutlin-3 (10 μM), Cisplatin (7.5 μM), UV rays (20 J/m^2^), or untreated cells. Relative expression ratios were measured respect to the sample transfected with the empty pcDNA3-HA and normalized by the expression levels of GAPDH. Data are shown as the average with standard deviation of 3 independent experiments (* p-value < 0.05; ** p-value < 0.01).

**Supplementary Fig. 10. (A)** Densitometry analysis of the average of 3 immunoblot experiments, one of which for cell line is presented in Figure 5C. The relative amounts of proteins, reported in the table, have been calculated normalizing to β-actin and calibrating to the respective control. The error bars represent the standard deviation. **(B)** Cell proliferation by MTT reduction assay in MCF7 transfected with empty pcDNA3-HA or the recombinant pcDNA3-HA-TRIM8 vectors and treated with Nutlin-3 (10 μM), Cisplatin (7.5 μM), UV rays (20 J/m^2^), or untreated cells. Data are shown as the average with standard deviation of 3 independent experiments (* p-value < 0.05; ** p-value < 0.01).

**Supplementary Fig. 11. (A)** WB of the indicated proteins in HCT116 transfected with control unspecific shRNA or TRIM8-specific shRNAs and treated with Nutlin-3 (10 μM), Cisplatin (7.5 μM), UV rays (20 J/m^2^), or untreated cells. Western blot of β-actin was conducted as control. **(B)** Expression levels of TRIM8 were measured by RT-qPCR in HCT116 cells, transfected with unspecific shRNA (control) or TRIM8-specific shRNAs and treated with Nutlin-3 (10 μM), Cisplatin (7.5 μM), UV rays (20 J/m^2^), or untreated cells. Relative expression was measured respect to the sample transfected with the control unspecific shRNA and normalized by the expression level of GAPDH. Data are shown as the average with standard deviation of at least 3 independent experiments (* p-value < 0.05; ** p-value < 0.01). **(C)** Cell proliferation by MTT reduction assay in HCT116 transfected with control unspecific shRNA or TRIM8-specific shRNAs and treated with Nutlin-3 (10 μM), Cisplatin (7.5 μM), UV rays (20 J/m^2^), or untreated cells. Data are shown as the average with standard deviation of 3 independent experiments (* p-value < 0.05). **(D)** WB of the indicated proteins in HCT116 transfected with empty pcDNA3-HA or the recombinant pcDNA3-HA-TRIM8 vectors and treated with Nutlin-3 (10 μM), Cisplatin (7.5 μM), UV rays (20 J/m^2^), or untreated cells. WB of β-actin was conducted as control. **(E)** Cell proliferation by MTT reduction assay in HCT116 transfected with empty pcDNA3-HA or the recombinant pcDNA3-HA-TRIM8 vectors, and treated with Nutlin-3 (10 μM), Cisplatin (7.5 μM), UV rays (20 J/m^2^), or untreated cells. Measurements were normalized respectively to the sample transfected with the control shRNAs or with the empty pcDNA3. Data are shown as the average with standard deviation of 3 independent experiments (* p-value < 0.05; ** p-value < 0.01).

**Supplementary Fig. 12. (A)** Expression levels of p21, ADA and CCND3 were measured by RTqPCR in the HCT116 cells transfected with control unspecific shRNA or TRIM8-specific shRNAs and treated with Nutlin-3 (10 μM), Cisplatin (7.5 μM), UV rays (20 J/m^2^), or untreated cells. Relative expression ratios were measured respect to the sample transfected with the control unspecific shRNA and normalized by the expression levels of GAPDH. Data are shown as the average with standard deviation of 3 independent experiments (* p-value < 0.05; ** pvalue < 0.01). **(B)** Expression levels of p21, ADA and CCND3 were measured by RT-qPCR in the HCT116 cells transfected with empty pcDNA3-HA or the recombinant pcDNA3-HA-TRIM8 vectors and treated with Nutlin-3 (10 μM), Cisplatin (7.5 μM), UV rays (20 J/m^2^), or untreated cells. Relative expression ratios were measured respect to the sample transfected with the empty pcDNA3-HA and normalized by the expression levels of GAPDH. Data are shown as the average with standard deviation of 3 independent experiments (* p-value < 0.05; ** p-value < 0.01).

**Supplementary Fig. 13. (A)** WB of the indicated proteins in U2OS transfected with control unspecific shRNA or TRIM8-specific shRNAs and treated with Nutlin-3 (10 μM), Cisplatin (7.5 μM), UV rays (20 J/m^2^), or untreated cells. Western blot of β-actin was conducted as control. **(B)** Expression levels of TRIM8 were measured by RT-qPCR in U2OS cells, transfected with unspecific shRNA (control) or TRIM8-specific shRNAs and treated with Nutlin-3 (10 μM), Cisplatin (7.5 μM), UV rays (20 J/m^2^), or untreated cells. Relative expression was measured respect to the sample transfected with the control unspecific shRNAs and normalized by the expression level of GAPDH. Data are shown as the average with standard deviation of 3 independent experiments (* p-value < 0.05; ** p-value < 0.01). **(C)** Cell proliferation by MTT reduction assay in U2OS transfected with control unspecific shRNA or TRIM8-specific shRNAs and treated with Nutlin-3 (10 μM), Cisplatin (7.5 μM), UV rays (20 J/m^2^), or untreated cells. Data are shown as the average with standard deviation of at least 3 independent experiments (* p-value < 0.05; ** pvalue < 0.01). **(D)** WB of the indicated proteins in U2OS transfected with empty pcDNA3-HA or the recombinant pcDNA3-HA-TRIM8 vectors and treated with Nutlin-3 (10 μM), Cisplatin (7.5 μM), UV rays (20 J/m^2^), or untreated cells. WB of β-actin was conducted as control. **(E)** Cell proliferation by MTT reduction assay in U2OS transfected with empty pcDNA3-HA or the recombinant pcDNA3-HA-TRIM8 vectors, and treated with Nutlin-3 (10 μM), Cisplatin (7.5 μM), UV rays (20 J/m^2^), or untreated cells. Measurements were normalized respectively to the sample transfected with the control shRNAs or with the empty pcDNA3. Data are shown as the average with standard deviation of 3 independent experiments (* p-value < 0.05; ** p-value < 0.01).

**Supplementary Fig. 14. (A)** Expression levels of p21, ADA and CCND3 were measured by RTqPCR in the U2OS cells transfected with control unspecific shRNAs (control) or TRIM8-specific shRNAs and treated with Nutlin-3 (10 μM), Cisplatin (7.5 μM), UV rays (20 J/m^2^), or untreated cells. Relative expression ratios were measured respect to the sample transfected with the control unspecific shRNAs and normalized by the expression levels of GAPDH. Data are shown as the average with standard deviation of 3 independent experiments (* p-value < 0.05; ** pvalue < 0.01). **(B)** Expression levels of p21, ADA and CCND3 were measured by RT-qPCR in the U2OS cells transfected with empty pcDNA3-HA or the recombinant pcDNA3-HA-TRIM8 vectors and treated with Nutlin-3 (10 μM), Cisplatin (7.5 μM), UV rays (20 J/m^2^), or untreated cells. Relative expression ratios were measured respect to the sample transfected with the empty pcDNA3-HA and normalized by the expression levels of GAPDH. Data are shown as the average with standard deviation of 3 independent experiments (* p-value < 0.05; ** p-value < 0.01).
